# Insulin-like growth factor type 1 deficiency in a Moroccan patient with de novo inverted duplication 9p24p12 and developmental delay: a case report

**DOI:** 10.1186/s13256-016-0830-x

**Published:** 2016-05-13

**Authors:** Saadia Amasdl, Abdelhafid Natiq, Siham Chafai Elalaoui, Aziza Sbiti, Thomas Liehr, Abdelaziz Sefiani

**Affiliations:** 1 Centre de Génomique Humaine, Faculté de Médecine et de Pharmacie, Université Mohammed V Souissi, Rabat, Morocco; 2 Département de Génétique Médicale, Institut National d’Hygiène, Rabat, Morocco; 3 Faculté des Sciences, Université Mohammed V, Agdal, Rabat, Morocco; 4 Institute of Human Genetics, University Hospital Jena, Jena, Germany

**Keywords:** 9p duplication, IGF-1 deficiency, Multicolor banding, *IGFBPL1*

## Abstract

**Background:**

9p duplication is a structural chromosome abnormality, described in more than 150 patients to date. In most cases the duplicated segment was derived from a parent being a reciprocal translocation carrier. However, about 15 cases with de novo 9p duplication have been reported previously. Clinically, this condition is characterized by mental retardation, short stature, developmental delay, facial dysmorphism, hand and toe anomalies, heart defects and/or ocular manifestations.

**Case presentation:**

We report here the case of a 2-year-old Moroccan girl with a de novo duplication of 9p24 to p12. Clinical manifestations included failure to thrive, psychomotor delay, microcephaly, dysmorphic features, equinus feet, and umbilical hernia. Further clinical investigations showed an insulin-like growth factor type 1 deficiency. Banding cytogenetics identified a derivative chromosome 9, with an abnormally elongated short arm. Molecular cytogenetics based on multicolor banding probes characterized an inverted duplication 9p24 to p12 involving several genes especially an insulin-like growth factor binding protein named insulin-like growth factor binding protein-like 1, which seemed to be overexpressed, leading to the insulin-like growth factor deficiency in our patient.

**Conclusions:**

This study showed that insulin-like growth factor type 1 deficiency can be another feature of 9p duplication, suggesting a likely involvement of insulin-like growth factor binding protein-like 1 overexpression in growth delay. However, further studies of the gene expressions are needed to better understand the phenotype-karyotype correlations.

## Background

9p duplication is a structural chromosome abnormality first described by Rethoré and colleagues [1]. To date more than 150 cases have been reported; however, the duplication is often due to a parental reciprocal balanced translocation, that is, beside the 9p duplication another chromosomal region is present in one copy only [2]. De novo duplications of this chromosomal region have been described in only about 15 cases, up to now [3–9]. Nonetheless, clinically this is a recognizable spectrum with specific major features like failure to thrive, psychomotor delay, mental retardation, craniofacial abnormalities (microcephaly, downslanting palpebral fissures, deep-set eyes, hypertelorism, bulbous nose, short philtrum, downturned corners of the mouth, short neck), digital abnormalities (fifth finger clinodacyly, brachydacyly, dysplastic nails), as well as skeletal malformations [10]. Here, we describe a case of a patient admitted for different clinical problems including insulin-like growth factor type 1 (IGF-1) deficiency with partial trisomy of 9p.

## Case presentation

Our patient, a 2-year-old girl, was the third child of healthy nonconsanguineous parents of Moroccan origin, born at term after an uneventful 39-week gestation and normal delivery; she was admitted for genetic evaluation because of psychomotor delay and failure to thrive. Her birth weight was 2500 g (3rd centile), length was 46 cm (3rd centile), and head circumference was 32 cm (3rd centile). Her family history was unremarkable for developmental delay or recurrent miscarriages. The proposita sat at 18 months, but her walking and language acquisition were delayed. On clinical examination, her length, weight, and head circumference at 2 years old were as follows: 68 cm (<3rd centile), 8 kg (<3rd centile) and 44 cm (<3rd centile). She had mild dysmorphic features similar to that of the 9p duplication syndrome. She had hypertelorism, deep-set eyes, broad nasal bridge and bulbous nasal tip, short philtrum, downturned mouth, retrognathia, and short neck. Additional findings included large anterior fontanelle, fifth finger clinodactyly, left equinus foot, and umbilical hernia. Further evaluation revealed growth hormone deficiency with decreased serum level of IGF-1, estimated at 47 ng/mL; whereas normal values are between 51 and 327 ng/mL. Magnetic resonance imaging (MRI) scan of pituitary gland was normal.

### Cytogenetic analysis

**Fig. 1:**
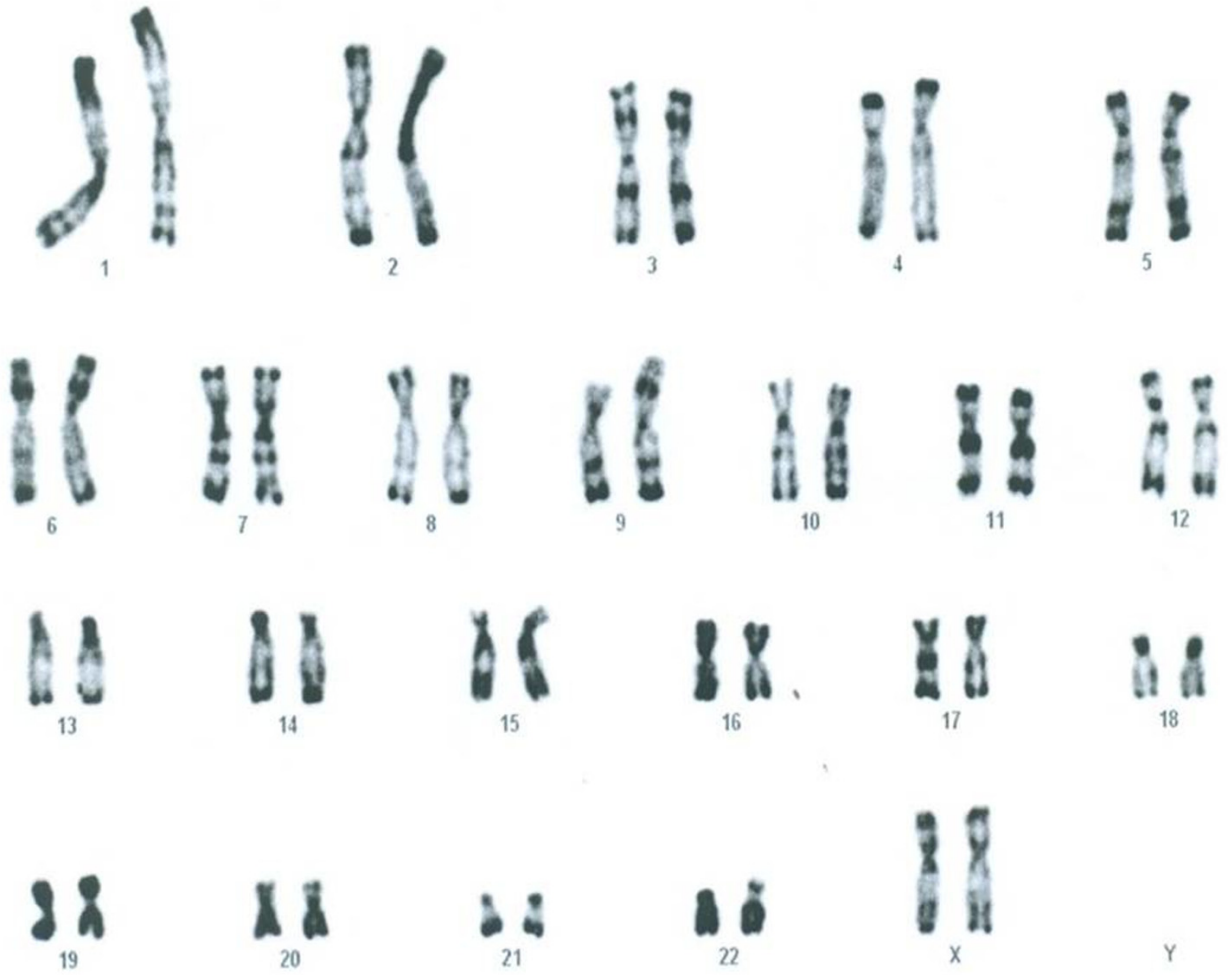
RHG banding (400-band resolution) shows a derivative 9 chromosome with an abnormally elongated p arm

Chromosomal analysis was performed on cultured peripheral lymphocytes of our patient and her parents according to standard methods. R banding at the resolution level of 400 bands was performed, as well as C banding after barium hydroxide treatment. RHG analysis (R-banding of human chromosomes by heat denaturation and Giemsa staining) showed a derivative of chromosome 9 with a 9p arm notably expanded. The extra band was C banding negative, thus excluding pericentric inversion of the 9qh region. This was interpreted as representing either a 9p duplication or some other rearrangement. Since parental karyotypes were both normal, our patient’s karyotype was designated as 46,XX,der(9)?dn (Fig. 1).

Thereafter, fluorescence *in situ* hybridization (FISH) test was done, applying multicolor banding probe set for chromosome 9 [11]. Probe labeling, hybridization post washing, signal detection, and image acquisition were performed as previously reported [12, 13]. For characterization of the heteromorphic patterns of chromosome 9, further probe set was applied [14, 15].

### Cytogenetic results

**Fig. 2:**
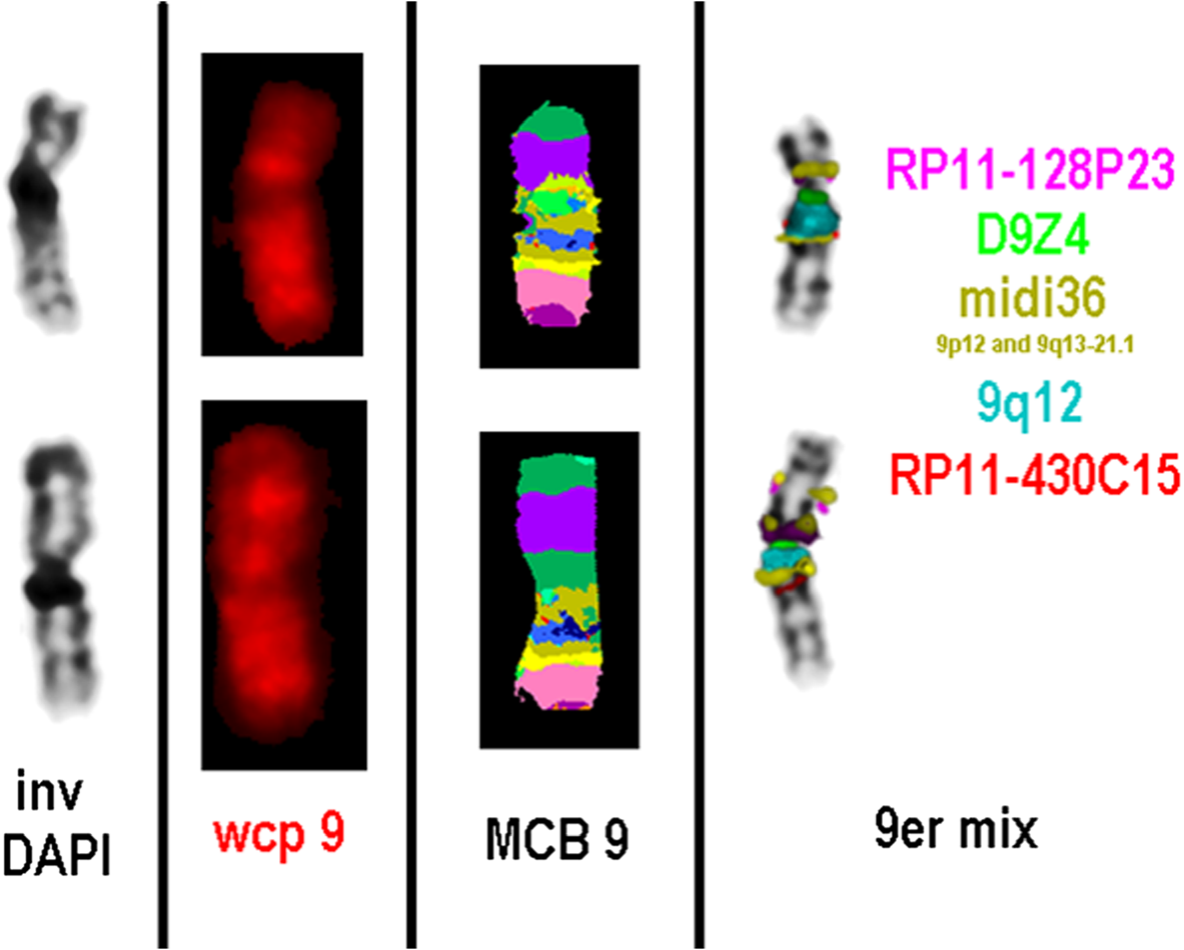
Fluorescence *in situ* hybridization result after application of WCP 9 and MCB9, 9 alpha-satellite probe, and satellite III probe, midi36 probe specific for 9p12 and 9q13-21.1. RP11-128P23 in 9p12 and RP11-430C15 in 9q13 confirmed that the region 9p24 to 9p12 was duplicated and inserted inverted in 9p12~13

FISH experiments identified the extra segment as a duplication of 9p24 to 9p12. The karyotype could be characterized after the application of the probes mentioned above. There was a partial trisomy 9p24 to 9p12. The region 9p24 to 9p12 was duplicated and inserted inverted in 9p12~13 (Fig. 2). The final karyotype was designated as follows: 46,XX,der(9)(pter->p12~13::p12->p24::p12~13->qter)dn.

## Discussion

Even though 9p duplication is a well-described syndrome, there are only few cases where the duplicated fragment is not inherited due to a parental balanced translocation. Table 1 shows clinical findings of patients reported in the literature with pure de novo 9p24p12 duplication [4, 16, 17]. The phenotype of our patient was consistent with the clinical spectrum described in the other comparable cases. However, she lacked hypoplastic nails, brachydactyly and strabismus. Only our patient presented with umbilical hernia, which is an uncommon finding and rarely reported [18]. Short stature has been reported infrequently in these patients, and IGF-1 deficiency specifically has only been seen twice before [19, 20].

**Table 1:** Clinical features in patients with de novo 9p12p24 duplication

First author of reference	Duplication 9p	Congenital abnormalities
Our patient	p12-p24 inverted	- Microcephaly, large anterior fontanel - Short stature, psychomotor delay - Hypertelorism, deep-set eyes, down-set ears, bulbous nose tip, broad nasal bridge, short philtrum, downturned corners of the mouth, retrognathia, short neck - Fifth finger clinodactyly, left foot equinus - Umbilical hernia. - Growth hormone deficiency
Cuoco *et al*., 1982 [16]	p12-p24 tandem	- **Short stature**, **psychomotor retardation**, puberty delay, Mental retardation - **Hypertelorism, deep-set eyes**, convergent strabismus, antimongoloid slant of eyes, malformed protruding ears, **downturned corners of the mouth**, dental malocclusion - **Fifth finger clinodactyly**, bilateral hypoplasia of the fourth metacarpal bone, hypoplastic nails, knee and elbow valgus, delayed bone age
Motegi *et al*., 1985 [17]	p12-p24 tandem	- **Microcephaly**, brachycephaly, **large anterior fontanelle** - **Short stature** - **Hypertelorism,** antimongoloid slant of eyes, cup-shaped ears, **prominent nasal bridge, bulbous nose**, **downturned corners of the mouth,** cleft lip and palate, - Small hands and feet, hypoplastic nails
Tsezou *et al*., 2000 [4]	p12-p24 tandem	- Brachycephaly - **Psychomotor delay** - High forehead, **hypertelorism**, epicanthus, **deep-set eyes,** cup-shaped ears, **bulbous nasal tip**, thin upper lip, **downturned corners of the mouth**, micro-retrognathia, short broad neck - Syndactyly of the third and fourth fingers, syndactyly of the second to fourth toes, hypoplastic nails - Widely spaced nipples, left cerebellar hypoplasia
Case 1		
Case 2		
	p12-p24 inverted	- Brachycephaly - **Psychomotor delay** - Frontal bossing**, hypertelorism**, epicanthus, **deep-set eyes, strabismus,** cup-shaped ears, **bulbous nasal tip**, **downturned corners of the mouth**, short broad neck - Widely spaced nipples - Short upper lip, short thumbs, transverse single palmar crease

Words in bold represents features in common with our patient

FISH-based banding methods allowed us to characterize the 9 chromosome rearrangement as a pure inverted 9p spanning from 9p24 to 9p12. This variant is rare and has been reported only once before [4]. Despite our patient carrying one of the largest duplicated 9p segments, there is a remarkable consistency in the phenotype especially in the facial and digital anomalies. This can be explained not only by the fact that 9p chromosome is relatively poor in genes [10], but also the duplicated segment encompasses critical region defined as 9p22 as well [2].

Based on the National Center for Biotechnology Information (NCBI) Map Viewer (www.ncbi.nlm.nih.gov/mapview/), the duplicated region in our patient spans 39 Mb, involving 434 with only 29 annotated genes. Interestingly, insulin-like growth factor binding protein-like 1 (*IGFBPL1*) gene localized in 9p13.1, and encoding a protein belonging to the insulin-like growth factor binding protein (IGFBP) family. These proteins bind to insulin-like growth factors (IGFs), and sometimes modulate the growth effects of IGFs. *IGFBPL1* was found to be most closely related to *IGFBP-7* with 52 % amino acid homology and 43 % amino acid identity, and shares a similar domain structure [21]. Previous study has demonstrated that *IGFBP-7* acts as an IGF-1/2 antagonist which can block insulin-like growth factor 1 receptor (IGF1R) activation by binding to the receptor itself [22]. Thereby, the homology between *IGFBP-1* and *IGFBP-7* suggests that the overexpression of the *IGFBP-1* gene may explain the IGF-1 deficiency and therefore the growth delay described in 9p duplication.

## Conclusions

This study showed that IGF-1 deficiency can be another feature of 9p duplication, suggesting a possible role of *IGFBPL1* overexpression in growth delay. However, further studies of the gene expressions are needed to better understand the phenotype-karyotype correlations.

## Consent

Written informed consent was obtained from the patient’s legal guardian(s) for publication of this case report and any accompanying images. A copy of the written consent is available for review by the Editor-in-Chief of this journal.

## Abbreviations

FISH: Fluorescence *in situ* hybridization
IGF-1: Insulin-like growth factor type 1
IGF1R: Insulin-like growth factor type 1 receptor
IGFBP: Insulin-like growth factor binding protein
IGFBPL1: Insulin-like growth factor binding protein-like 1

## References

[1] Rethoré MO, Larget-Piet L, Abonyi D, Boeswillwald M, Berger R, Carpentier S, et al. 4 cases of trisomy for the short arm of chromosome 9. Individualization of a new morbid entity. Ann Genet. 1970;13:217–32. 5313386

[2] Haddad BR, Lin AE, Wyandt H, Milunsky A. Molecular cytogenetic characterization of the first familial case of partial 9p duplication (p22p24). J Med Genet. 1996;33:1045–7. 10.1136/jmg.33.12.1045 PMC1050821 9004142

[3] Sanlaville D, Baumann C, Lapierre JM, Romana S, Collot N, Cacheux V, et al. De novo inverted duplication 9p21pter involving telomeric repeated sequences. Am J Med Genet. 1999;83:125–31. 10.1002/(sici)1096-8628(19990312)83:2<125::aid-ajmg8>3.0.co;2-0 10190483

[4] Tsezou A, Kitsiou S, Galla A, Petersen MB, Karadima G, Syrrou M, et al. Molecular cytogenetic characterization and origin of two de novo duplication 9p cases. Am J Med Genet. 2000;91:102–6. 10.1002/(sici)1096-8628(20000313)91:2<102::aid-ajmg4>3.0.co;2-5 10748406

[5] Bonaglia MC, Giorda R, Carrozzo R, Roncoroni ME, Grasso R, Borgatti R, et al. 20-Mb duplication of chromosome 9p in a girl with minimal physical findings and normal IQ: narrowing of the 9p duplication critical region to 6 Mb. Am J Med Genet. 2002;112:154–9. 10.1002/ajmg.10699 12244548

[6] Krepischi-Santos AC, Vianna-Morgante AM. Disclosing the mechanisms of origin of de novo short-arm duplications of chromosome 9. Am J Med Genet A. 2003;117A:41–6. 10.1002/ajmg.a.10634 12548739

[7] Hulick PJ, Noonan KM, Kulkarni S, Donovan DJ, Listewnik M, Ihm C, et al. Cytogenetic and array-CGH characterization of a complex de novo rearrangement involving duplication and deletion of 9p and clinical findings in a 4-month-old female. Cytogenet Genome Res. 2009;126:305–12. 10.1159/000251966 PMC3711006 20068300

[8] Al Achkar W, Wafa A, Moassass F, Liehr T. Partial trisomy 9p22 to 9p24.2 in combination with partial monosomy 9pter in a Syrian girl. Mol Cytogenet. 2010;3:18. 10.1186/1755-8166-3-18 PMC2958875 20920324

[9] Chen CP, Lin SP, Su YN, Chern SR, Tsai FJ, Chen WL, et al. Self-injurious behavior associated with trisomy 9p (9p13.1 --> p24.3). Genet Couns. 2011;22:327–31. 22029177

[10] Guilherme RS, Meloni VA, Perez AB, Pilla AL, de Ramos MA, Dantas AG, et al. Duplication 9p and their implication to phenotype. BMC Med Genet. 2014;15:142. 10.1186/s12881-014-0142-1 PMC4411943 25526829

[11] Weise A, Mrasek K, Fickelscher I, Claussen U, Cheung SW, Cai WW, et al. Molecular definition of high-resolution multicolor banding probes: first within the human DNA sequence anchored FISH banding probe set. J Histochem Cytochem. 2008;56:487–93. 10.1369/jhc.2008.950550 PMC2324187 18256020

[12] Liehr T, Heller A, Starke H, Claussen U. FISH banding methods: applications in research and diagnostics. Expert Rev Mol Diagn. 2002;2:217–25. 10.1586/14737159.2.3.217 12050860

[13] Chudoba I, Hickmann G, Friedrich T, Jauch A, Kozlowski P, Senger G. mBAND: a high resolution multicolor banding technique for the detection of complex intrachromosomal aberrations. Cytogenet Genome Res. 2004;104:390–3. 10.1159/000077521 15162070

[14] Starke H, Seidel J, Henn W, Reichardt S, Volleth M, Stumm M, et al. Homologous sequences at human chromosome 9 bands p12 and q13-21.1 are involved in different patterns of pericentric rearrangements. Eur J Hum Genet. 2002;10:790–800. 10.1038/sj.ejhg.5200889 12461685

[15] Kosyakova N, Grigorian A, Liehr T, Manvelyan M, Simonyan I, Mkrtchyan H, et al. Heteromorphic variants of chromosome 9. Mol Cytogenet. 2013;6:14. 10.1186/1755-8166-6-14 PMC3626942 23547710

[16] Cuoco C, Gimelli G, Pasquali F, Poloni L, Zuffardi O, Alicata P, et al. Duplication of the short arm of chromosome 9.Analysis of five cases. Hum Genet. 1982;61:3–7. 10.1007/BF00291321 7129421

[17] Motegi T, Watanabe K, Nakamura N, Hasegawa T, Yanagawa Y. De novo tandem duplication 9p (p12-p24) with normal GALT activity in red cells. J Med Genet. 1985;22:64–6. 10.1136/jmg.22.1.64 PMC1049380 2984424

[18] Schinzel A. Catalogue of unbalanced chromosome aberrations in man. 2nd ed. New York: W de Gruyter Berlin; 2001.

[19] Stagi S, Lapi E, Seminara S, Guarducci S, Pantaleo M, Giglio S, et al. Long-term auxological and endocrinological evaluation of patients with 9p trisomy: a focus on the growth hormone-insulin-like growth factor-I axis. BMC Endocr Disord. 2014;14:3. 10.1186/1472-6823-14-3 PMC3893409 24397778

[20] Fujita H, Shimazaki M, Takeuchi T, Hayakawa Y, Oura T. 47,+(9q-) in unrelated three children with plasma growth hormone deficiency. Hum Genet. 1976;31:271–82. 10.1007/BF00270857 955626

[21] Cai Z, Chen HT, Boyle B, Rupp F, Funk WD, Dedera DA. Identification of a novel insulin-like growth factor binding protein gene homologue with tumor suppressor like properties. Biochem Biophys Res Commun. 2005;331:261–6. 10.1016/j.bbrc.2005.03.163 15845387

[22] Evdokimova V, Tognon CE, Tania B, Yang W, Krutikov K, Pollak M, et al. IGFBP7 binds to the IGF-1 receptor and blocks its activation by insulin-like growth factors. Sci Signal. 2012;5:ra92. 10.1126/scisignal.2003184 23250396

